# Crystal structure of poly[[di­aqua­tetra-μ_2_-cyanido-iron(II)platinum(II)] acetone disolvate]

**DOI:** 10.1107/S2056989019012945

**Published:** 2019-09-27

**Authors:** Iryna S. Kuzevanova, Dina D. Naumova, Kateryna V. Terebilenko, Sergiu Shova, Il’ya A. Gural’skiy

**Affiliations:** a Department of General and Inorganic Chemistry, National Technical University of Ukraine "Igor Sikorsky Kyiv Polytechnic Institute", Prosp. Peremogy 37, Kyiv 03056, Ukraine; b Department of Chemistry, Taras Shevchenko National University of Kyiv, Volodymyrska St. 64, Kyiv 01601, Ukraine; c Department of Inorganic Polymers, "Petru Poni" Institute of Macromolecular Chemistry, Romanian Academy of Science, Aleea Grigore Ghica Voda 41-A, Iasi 700487, Romania; d UkrOrgSyntez Ltd, Chervonotkatska St., 67, Kyiv 02094, Ukraine

**Keywords:** crystal structure, Hofmann clathrate, iron(II)

## Abstract

In the title polymeric complex, {[FePt(CN)_4_(H_2_O)_2_]·2C_3_H_6_O}_*n*_, the Fe^II^ cation has an octa­hedral [FeN_4_O_2_] geometry being coordinated by two water mol­ecules and four cyanide anions. The Pt cation is located on an inversion centre and has a square-planar coordination environment formed by four cyanide groups. The tetra­cyano­platinate anions bridge the Fe^II^ cations to form infinite two-dimensional layers that propagate in the *bc* plane. Two guest mol­ecules of acetone per Fe^II^ are located between the layers. These guest acetone mol­ecules inter­act with the coordinated water mol­ecules by O—H⋯O hydrogen bonds.

## Abstract 2

In the title polymeric complex, the tetra­cyano­platinate anions bridge the Fe^II^ cations to form infinite two-dimensional layers that propagate along the *bc* plane. Two guest mol­ecules of acetone per Fe^II^ are located between the layers. These guest acetone mol­ecules inter­act with the coordinated water mol­ecules by O—H⋯O hydrogen bonds.

## Chemical context   

Hofmann clathrates and their analogues form one the most famous families of compounds that are able to incorporate guest mol­ecules. The first clathrate was obtained by Hofmann and Küspert in 1897 (Hofmann & Küspert, 1897 ▸) and was of composition [Ni(NH_3_)_2_Ni(CN)_4_]·2C_6_H_6_. It was a 2D coord­in­ation compound formed by infinite cyano­metallic layers that propagate along the *ab* plane. The 2D system was supported by ammine axial ligands, and guest mol­ecules of benzene were trapped between the layers.

Later, by slight modifications of the chemical composition, several analogous compounds were obtained, leading to the creation of a new class of coordination materials. The first modification was the substitution of nickel with other transition metals, as well as the introduction of other small aromatic guest mol­ecules that resulted in the creation of compounds with the general formula [*M*(NH_3_)_2_ *M*′(CN)_4_]·2*G* (where *M* = Mn, Fe, Co, Ni, Cu, Zn, Cd, *M*′ = Ni, Pd, Pt and *G* = benzene, pyrrole, thio­phene, aniline, biphenyl, *etc*.; Iwamoto, 1996 ▸). The second modification of the Hofmann clathrate was the substitution of square-planar {*M*′(CN)_4_}^2−^ anions with tetra­hedral ({*M*′(CN)_4_}^2−^, *M*′ = Cd, Hg; Arcís-Castillo *et al.*, 2013 ▸), linear ({*M*′(CN)_2_}^−^, *M*′ = Cu, Ag, Au; Gural’skiy, Golub *et al.*, 2016 ▸; Gural’skiy, Shylin *et al.*, 2016 ▸), octa­hedral ({*M*′(CN)_6_}^3−^, *M*′ = Co, Cr; Dommann *et al.*, 1990 ▸) and even dodeca­hedral ({*M*′(CN)_8_}^4−^, *M*′ = W, Nb; Ohkoshi *et al.*, 2013 ▸) fragments. Additionally, a very important modification was made by the introduction of other organic ligands instead of ammonia. For example, by the introduction of pyridine, the first Fe^II^-based clathrate [Fe(py)_2_{Pt(CN)_4_}] exhibiting spin-crossover (SCO) behaviour was obtained by Kitazawa *et al.* (1996 ▸). At the same time, the introduction of various bidentate ligands such as pyrazine (Niel *et al.*, 2001 ▸; Gural’skiy, Shylin *et al.*, 2016 ▸), pyrimidine (Agustí *et al.*, 2008 ▸), bis­(4-pyrid­yl)acetyl­ene (Agustí *et al.*, 2008 ▸) and others allowed the formation of 3D SCO networks.

Additionally, the characteristics of spin transition in coordination compounds are known to be extremely sensitive to any changes in the chemical environment. As Hofmann clathrate analogues are very easy to modulate, numerous SCO complexes with very different temperatures, abruptnesses and hystereses of SCO were obtained. Moreover, the ability of Hofmann clathrate analogues to incorporate guest mol­ecules provided SCO-based chemical sensors (Ohba *et al.*, 2009 ▸).
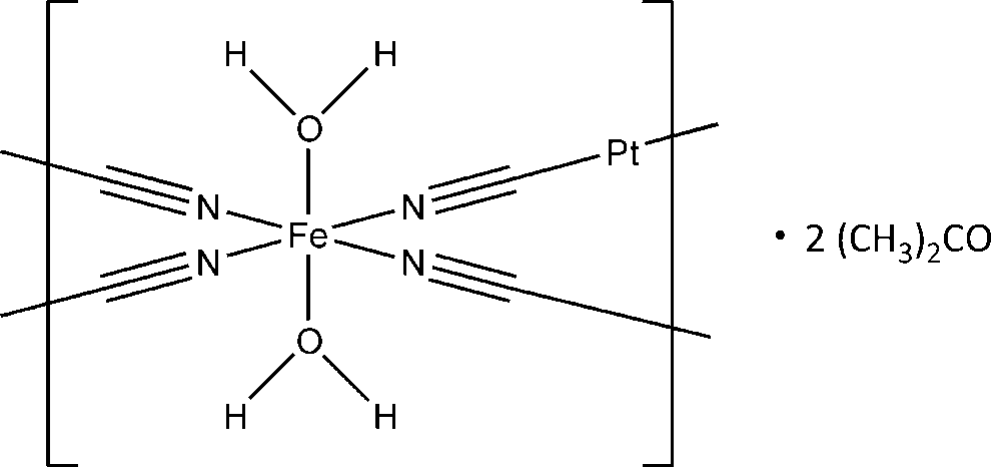


Herein we present a new Fe–Pt Hofmann clathrate analogue [Fe(H_2_O)_2_{Pt(CN)_4_}]·2(CH_3_)_2_CO.

## Structural commentary   

**Figure 1:**
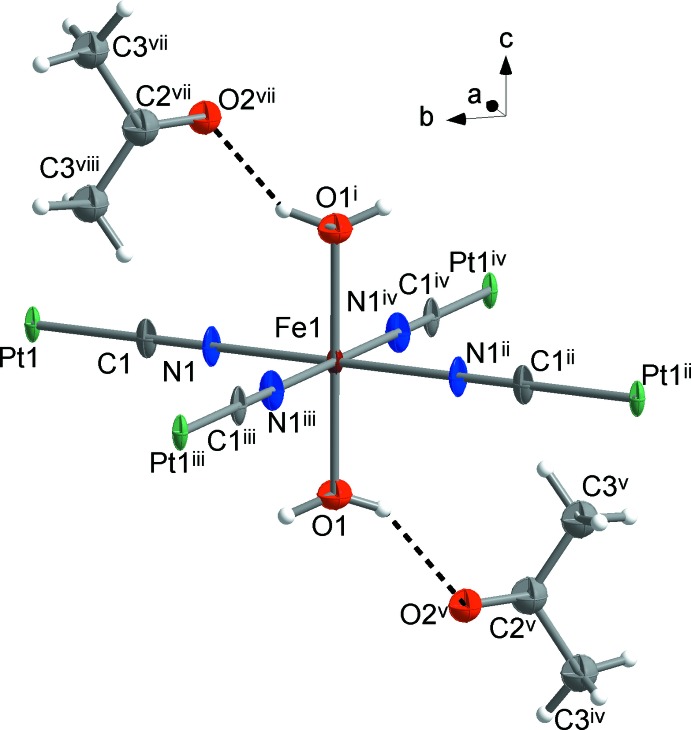
A fragment of the mol­ecular structure of the title compound showing the atom-labelling scheme. Displacement ellipsoids are drawn at the 50% probability level [symmetry codes: (i) *x*, −*y*, 1 − *z*; (ii) −*x*, −*y*, *z*; (iii) *x*, −*y*, *z*; (iv) −*x*, *y*, *z*; (v) −1 + *x*, −*y*, *z*; (vi) −1 + *x*, −*y*, −*z*; (vii) 1 − *x*, *y*, 1 + *z*; (viii) 1 − *x*, *y*, 1 − *z*].

The title compound crystallizes in the *P*4/*mmm* space group. The Fe^II^ cation has a [FeN_4_O_2_] coordination environment (Fig. 1 ▸) comprising four CN^−^ anions in the equatorial positions [Fe1—N1 = 2.158 (5) Å] and two water mol­ecules in the axial positions [Fe1—O1 = 2.130 (6) Å]. The Fe—O bonds are slightly shorter than the Fe–N bonds, thus leading to a compressed octa­hedral geometry. Judging by the bond length, the Fe^II^ cation is in a high-spin state at the experimental temperature (180 K). This is corroborated by the presence of H_2_O mol­ecules in the coordination sphere of Fe^II^. The cyanide anions connect the Fe^II^ and Pt^II^ cations into infinite two-dimensional layers. The Pt^II^ cation is located at a fourfold rotation axis and possesses a square-planar geometry [Pt1—C1 = 1.993 (6) Å, C1—Pt–C1 = 90°]. Thanks to the tetra­gonal symmetry of the crystalline compound, no deviation from an ideal octa­hedron is observed for Fe^II^, Σ|90 – θ| = 0°, where θ is the N—Fe—N or O—Fe—N angles. Additionally, the compound incorporates two guest mol­ecules of acetone per Fe^II^ centers.

## Supra­molecular features   

**Figure 2:**
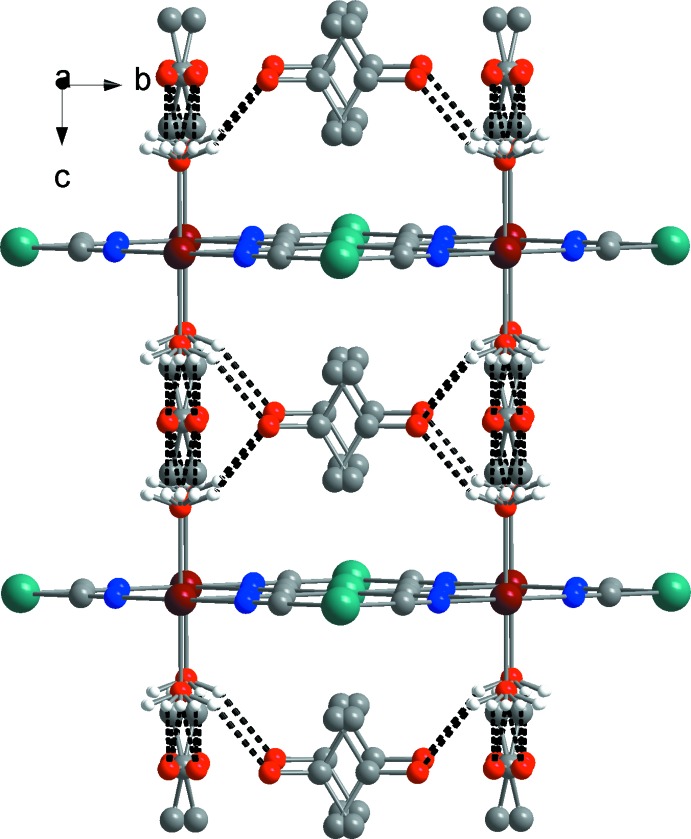
View of the crystal structure of the title compound in the *bc* plane showing the two-dimensional cyano­metallic layers. Hydrogen bonds are shown as dashed lines. Acetone H atoms are omitted for clarity.

**Figure 3:**
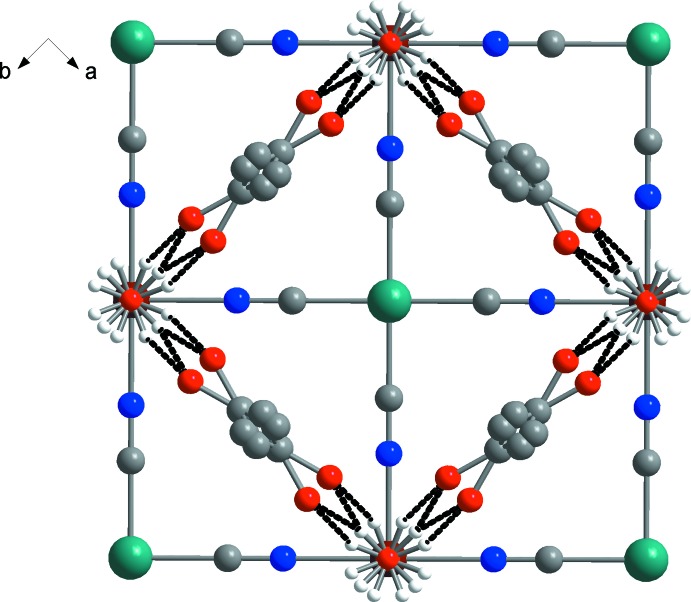
View of the structure of the title compound in the *ab* plane showing the distortion of the acetone guest mol­ecules. Hydrogen bonds are shown as dashed lines. Acetone H atoms are omitted for clarity.

The crystalline structure is connected by bridging tetra­cyano­platinate moieties, which form a two-dimensional grid that propagates along the *ab* plane (Fig. 2 ▸). As imposed by symmetry, no deviation from linearity for the Fe—N—C—Pt linkages is observed [Fe—N—C = 180°, N—C—Pt = 180°, C—Pt—C = 180°]. The distance between parallel cyano­metallic layers is 7.973 (6) Å. The guest acetone mol­ecules are located between the cyano­metallic layers. Each oxygen atom of the coordinated water mol­ecules inter­acts with acetone by O—H⋯O hydrogen bonds (Fig. 3 ▸, Table 1 ▸), creating a three-dimensional supramolecular framewor. The size of the available voids between the cyano­metallic layers allows the acetone mol­ecules to rotate freely, thus leading to disorder of the acetone mol­ecules over four positions.

## Database survey   

A survey of the Cambridge Structural Database (Version 5.38; Groom *et al.*, 2016 ▸) confirmed that the title compound has never been published before. It revealed 51 cyano­metallic structures of the general formula [*TM*(H_2_O)_2_{T*M*(CN)_4_}], where *TM* = any transition metal. There were also 19 hits for structures containing [Fe{Pt(CN)_4_}] fragments: refcodes: OVILEM, OVIRUI, OVIRUI01, OVIRUI02 and OVIRUI03 (Sciortino *et al.*, 2017 ▸), AMIJOX (Kucheriv *et al.*, 2016 ▸), BEDWEO and BEDWIS (Sciortino *et al.*, 2012 ▸), CEMYUQ (Mũnoz-Lara *et al.*, 2013 ▸), MUHMEI, MUHNAF, MUHNAF01, MUHNAF02, MUHPAH and MUHPAH01 (Martínez *et al.*, 2009 ▸), QADDUX (Sakaida *et al.*, 2016 ▸), QOJWIW and QOJWIW01 (Cobo *et al.*, 2008 ▸) and TURXIP (Ohtani *et al.*, 2013 ▸).

## Synthesis and crystallization   

Crystals of the title compound were obtained by slow diffusion (three layers) in a 3 ml tube. The first layer contained 19 mg (0.05 mmol) of K_2_[Pt(CN)_4_] in 0.5 ml of water. The middle layer contained 1.5 ml of a water:acetone (1:1) solution. The third layer contained 25 mg (0.05 mmol) of Fe(OTs)_2_·6H_2_O in 0.4 ml of acetone and 0.1 ml of water. The colourless crystals grew in the middle layer within three weeks and were kept in the mother solution prior to measurements.

## Refinement   

Crystal data, data collection and structure refinement details are summarized in Table 2 ▸. All hydrogen atoms were fixed at calculated positions and refined as riding with C—H = 0.96 Å and O–H = 0.84 Å, *U*_iso_(H) = 1.5*U*_iso_(C,O). The OH group and the idealized methyl group were refined as rotating.

## Supplementary Material

Crystal structure: contains datablock(s) I. DOI: 10.1107/S2056989019012945/tx2013sup1.cif

Additional supporting information:  crystallographic information; 3D view; checkCIF report

## Figures and Tables

**Table 1:** Hydrogen-bond geometry (Å, °)

*D*—H⋯*A*	*D*—H	H⋯*A*	*D*⋯*A*	*D*—H⋯*A*
O1—H1*A*⋯O2^i^	0.84	2.03	2.775 (11)	147
O1—H1*A*⋯O2^ii^	0.84	2.03	2.775 (11)	148
O1—H1*B*⋯O2^iii^	0.85	2.04	2.775 (11)	144
O1—H1*B*⋯O2^iv^	0.85	2.14	2.775 (11)	131

Symmetry codes: (i) 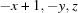; (ii) 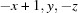; (iii) 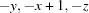; (iv) 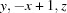.

**Table 2:** Experimental details

Crystal data	
Chemical formula	[FePt(CN)_4_(H_2_O)_2_]·2C_3_H_6_O
*M*_r_	507.21
Crystal system, space group	Tetragonal, *P*4/*m* *m* *m*
Temperature (K)	180
*a*, *c* (Å)	7.4802 (4), 7.9725 (11)
*V* (Å^3^)	446.09 (8)
*Z*	1
Radiation type	Mo *K*α
μ (mm^−1^)	8.66
Crystal size (mm)	0.05 × 0.05 × 0.02

Data collection	
Diffractometer	Rigaku Oxford Diffraction Xcalibur, Eos
Absorption correction	Multi-scan (*CrysAlis PRO*; Rigaku OD, 2015 ▸)
*T*_min_, *T*_max_	0.699, 1.000
No. of measured, independent and observed [*I* > 2σ(*I*)] reflections	1126, 361, 359
*R*_int_	0.037
(sin θ/λ)_max_ (Å^−1^)	0.682

Refinement	
*R*[*F* ^2^ > 2σ(*F* ^2^)], *wR*(*F* ^2^), *S*	0.025, 0.046, 1.04
No. of reflections	361
No. of parameters	34
H-atom treatment	H-atom parameters constrained
Δρ_max_, Δρ_min_ (e Å^−3^)	1.25, −1.06

Computer programs: *CrysAlis PRO* (Rigaku OD, 2015 ▸), *SHELXT* (Sheldrick, 2015*a* ▸), *SHELXL* (Sheldrick, 2015*b* ▸) and *OLEX2* (Dolomanov *et al.*, 2009 ▸).

## supplementary crystallographic information

### Crystal data

**Table:**

[FePt(CN)_4_(H_2_O)_2_]·2C_3_H_6_O	*D*_x_ = 1.888 Mg m^−^^3^
*M**_r_* = 507.21	Mo *K*α radiation, λ = 0.71073 Å
Tetragonal, *P*4/*m**m**m*	Cell parameters from 664 reflections
*a* = 7.4802 (4) Å	θ = 2.5–28.5°
*c* = 7.9725 (11) Å	µ = 8.66 mm^−^^1^
*V* = 446.09 (8) Å^3^	*T* = 180 K
*Z* = 1	Plate, clear colourless
*F*(000) = 240	0.05 × 0.05 × 0.02 mm

### Data collection

**Table:**

Rigaku Oxford Diffraction Xcalibur, Eos diffractometer	361 independent reflections
Radiation source: fine-focus sealed X-ray tube, Enhance (Mo) X-ray Source	359 reflections with *I* > 2σ(*I*)
Graphite monochromator	*R*_int_ = 0.037
Detector resolution: 8.0797 pixels mm^-1^	θ_max_ = 29.0°, θ_min_ = 2.6°
ω scans	*h* = −5→10
Absorption correction: multi-scan (CrysAlisPro; Rigaku OD, 2015)	*k* = −10→5
*T*_min_ = 0.699, *T*_max_ = 1.000	*l* = −10→5
1126 measured reflections	

### Refinement

**Table:**

Refinement on *F*^2^	Primary atom site location: dual
Least-squares matrix: full	Hydrogen site location: mixed
*R*[*F*^2^ > 2σ(*F*^2^)] = 0.025	H-atom parameters constrained
*wR*(*F*^2^) = 0.046	*w* = 1/[σ^2^(*F*_o_^2^) + (0.0197*P*)^2^] where *P* = (*F*_o_^2^ + 2*F*_c_^2^)/3
*S* = 1.03	(Δ/σ)_max_ < 0.001
361 reflections	Δρ_max_ = 1.25 e Å^−^^3^
34 parameters	Δρ_min_ = −1.06 e Å^−^^3^
0 restraints	

### Special details

**Table:**

Geometry. All esds (except the esd in the dihedral angle between two l.s. planes) are estimated using the full covariance matrix. The cell esds are taken into account individually in the estimation of esds in distances, angles and torsion angles; correlations between esds in cell parameters are only used when they are defined by crystal symmetry. An approximate (isotropic) treatment of cell esds is used for estimating esds involving l.s. planes.

### Fractional atomic coordinates and isotropic or equivalent isotropic displacement parameters (Å^2^)

**Table:**

	*x*	*y*	*z*	*U*_iso_*/*U*_eq_	Occ. (<1)
Pt1	0.500000	0.500000	0.500000	0.01400 (17)	
Fe1	0.000000	0.000000	0.500000	0.0104 (4)	
O1	0.000000	0.000000	0.2329 (8)	0.0276 (16)	
H1A	0.105309	0.000432	0.196819	0.041*	0.25
H1B	−0.043425	0.098216	0.196839	0.041*	0.125
C1	0.3116 (6)	0.3116 (6)	0.500000	0.0186 (15)	
N1	0.2040 (5)	0.2040 (5)	0.500000	0.0210 (13)	
C3	0.475 (9)	0.036 (8)	0.1546 (18)	0.08 (2)	0.25
H3A	0.473348	0.162844	0.175407	0.114*	0.25
H3B	0.531096	−0.023709	0.246770	0.114*	0.25
H3C	0.354004	−0.005924	0.142949	0.114*	0.25
O2	0.7276 (18)	0.043 (3)	0.000000	0.055 (5)	0.25
C2	0.574 (2)	0.000000	0.000000	0.055 (5)	0.5

### Atomic displacement parameters (Å^2^)

**Table:**

	*U*^11^	*U*^22^	*U*^33^	*U*^12^	*U*^13^	*U*^23^
Pt1	0.00585 (17)	0.00585 (17)	0.0303 (3)	0.000	0.000	0.000
Fe1	0.0068 (5)	0.0068 (5)	0.0177 (10)	0.000	0.000	0.000
O1	0.030 (2)	0.030 (2)	0.023 (4)	0.000	0.000	0.000
C1	0.0092 (18)	0.0092 (18)	0.037 (4)	0.003 (2)	0.000	0.000
N1	0.0121 (16)	0.0121 (16)	0.039 (4)	−0.005 (2)	0.000	0.000
C3	0.04 (4)	0.13 (5)	0.056 (9)	−0.03 (2)	0.013 (14)	−0.05 (2)
O2	0.019 (4)	0.109 (15)	0.036 (6)	−0.005 (10)	0.000	0.000
C2	0.019 (4)	0.109 (15)	0.036 (6)	−0.005 (10)	0.000	0.000

### Geometric parameters (Å, º)

**Table:**

Pt1—C1^i^	1.993 (6)	Fe1—N1^v^	2.158 (5)
Pt1—C1	1.993 (6)	Fe1—N1^vi^	2.158 (5)
Pt1—C1^ii^	1.993 (6)	Fe1—N1	2.158 (5)
Pt1—C1^iii^	1.993 (6)	C1—N1	1.138 (8)
Fe1—O1^iv^	2.130 (6)	C3—C2	1.46 (4)
Fe1—O1	2.130 (6)	O2—O2^vii^	0.64 (5)
Fe1—N1^iv^	2.158 (5)	O2—C2	1.19 (2)
			
C1^i^—Pt1—C1^ii^	90.0	O1^iv^—Fe1—N1^vi^	90.0
C1^ii^—Pt1—C1	90.0	N1^iv^—Fe1—N1^vi^	90.0
C1^i^—Pt1—C1	180.0	N1—Fe1—N1^vi^	90.0
C1^i^—Pt1—C1^iii^	90.0	N1^iv^—Fe1—N1^v^	90.0
C1^iii^—Pt1—C1	90.0	N1—Fe1—N1^iv^	180.0
C1^ii^—Pt1—C1^iii^	180.0	N1—Fe1—N1^v^	90.0
O1—Fe1—O1^iv^	180.0	N1^vi^—Fe1—N1^v^	180.0
O1—Fe1—N1	90.0	N1—C1—Pt1	180.0
O1—Fe1—N1^vi^	90.0	C1—N1—Fe1	180.0
O1^iv^—Fe1—N1^v^	90.0	O2^vii^—O2—C2	74.4 (12)
O1^iv^—Fe1—N1	90.0	O2^vii^—C2—C3	123 (2)
O1—Fe1—N1^v^	90.0	O2—C2—C3	116 (2)
O1—Fe1—N1^iv^	90.0	O2—C2—O2^vii^	31 (2)
O1^iv^—Fe1—N1^iv^	90.0		

Symmetry codes: (i) −*x*+1, −*y*+1, −*z*+1; (ii) *y*, −*x*+1, −*z*+1; (iii) −*y*+1, *x*, *z*; (iv) −*x*, −*y*, −*z*+1; (v) −*y*, *x*, *z*; (vi) *y*, −*x*, −*z*+1; (vii) *x*, −*y*, *z*.

### Hydrogen-bond geometry (Å, º)

**Table:**

*D*—H···*A*	*D*—H	H···*A*	*D*···*A*	*D*—H···*A*
O1—H1*A*···O2^viii^	0.84	2.03	2.775 (11)	147
O1—H1*A*···O2^ix^	0.84	2.03	2.775 (11)	148
O1—H1*B*···O2^x^	0.85	2.04	2.775 (11)	144
O1—H1*B*···O2^xi^	0.85	2.14	2.775 (11)	131

Symmetry codes: (viii) −*x*+1, −*y*, *z*; (ix) −*x*+1, *y*, −*z*; (x) −*y*, −*x*+1, −*z*; (xi) *y*, −*x*+1, *z*.
